# *In Vivo* Voltage-Sensitive Dye Imaging of Subcortical Brain Function

**DOI:** 10.1038/srep17325

**Published:** 2015-11-27

**Authors:** Qinggong Tang, Vassiliy Tsytsarev, Chia-Pin Liang, Fatih Akkentli, Reha S. Erzurumlu, Yu Chen

**Affiliations:** 1 Fischell Department of Bioengineering, University of Maryland, College Park, MD 20742 USA; 2 Department of Anatomy and Neurobiology, University of Maryland School of Medicine, Baltimore, MD 21201 USA

## Abstract

The whisker system of rodents is an excellent model to study peripherally evoked neural activity in the brain. Discrete neural modules represent each whisker in the somatosensory cortex (“barrels”), thalamus (“barreloids”), and brain stem (“barrelettes”). Stimulation of a single whisker evokes neural activity sequentially in its corresponding barrelette, barreloid, and barrel. Conventional optical imaging of functional activation in the brain is limited to surface structures such as the cerebral cortex. To access subcortical structures and image sensory-evoked neural activity, we designed a needle-based optical system using gradient-index (GRIN) rod lens. We performed voltage-sensitive dye imaging (VSDi) with GRIN rod lens to visualize neural activity evoked in the thalamic barreloids by deflection of whiskers *in vivo*. We stimulated several whiskers together to determine the sensitivity of our approach in differentiating between different barreloid responses. We also carried out stimulation of different whiskers at different times. Finally, we used muscimol in the barrel cortex to silence the corticothalamic inputs while imaging in the thalamus. Our results show that it is possible to obtain functional maps of the sensory periphery in deep brain structures such as the thalamic barreloids. Our approach can be broadly applicable to functional imaging of other core brain structures.

Localizing and real-time monitoring of neural activities evoked by peripheral stimulation are important steps in understanding the functional characteristics of neuronal circuits in the brain^1^. Imaging plays an important role in associating the activities of single neuron and cell ensembles with physiological and anatomical properties of the organism. Various optical imaging methods using either reflectance or fluorescence photons have shown to be very promising in functional brain mapping^2^. Intrinsic Optical Signal (IOS) imaging^3^,^4^, Diffuse Optical Imaging (DOI)^5^,^6^,^7^, Optical Coherence Tomography (OCT)^8^,^9^,^10^,^11^,^12^, Photoacoustic Tomography (PAT)^13^, as well as Multiphoton Microscopy (MPM)^14^,^15^, provide neuroscientists an opportunity to observe, noninvasively, the anatomy of the living brain, and to monitor its functions *in vivo*.

VSDi imaging offers an excellent opportunity to monitor the neural activity with high spatial and temporal resolution^16^,^17^,^18^,^19^. This method is based on the voltage-sensitive fluorescence probes, i.e., chemicals that change their optical features in response to the changes of the transmembrane electric field. The dye molecules bind to the neuronal membrane and convert changes in the transmembrane voltage into changes in fluorescence^2^. These changes are recorded by the optical imaging system and can be used for functional brain mapping.

Topographic neural maps of the sensory periphery have been known for a very long time. However, *in vivo* functional imaging of these maps is relatively new. The rodent whisker-barrel system is an excellent model to investigate the development, organization, function, and plasticity of mammalian sensory pathways. In this system, neuronal modules, representing single whiskers on the snout, form at the brainstem (barrelettes), thalamic (barreloids), and neocortical (barrels) levels^20^,^21^. Because of their ease of access, identification and close to surface location, the barrel fields have been the subject of numerous imaging studies with a variety of approaches^22^,^23^,^24^,^25^. In contrast to imaging of the neocortex, functional mapping of the subcortical structures using optical methods was technically challenging and limited^26^. Currently, miniature endoscopic probes offer a solution for deep brain imaging by overcoming the limited depth of intravital microscopy^27^. Taking advantage of this, we built a functional imaging system, which combines VSDi with a 1 mm-GRIN rod lens to monitor temporal and spatial dynamics of neural activities in the thalamus following whisker stimulation. We imaged neural activity in the ventral posteromedial (VPM) nucleus of the mouse thalamus responding to single whisker stimulation. With this approach, we were able to obtain a functional map of the thalamic barreloids.

## Results

### Cortical signal recording

**Figure 1:**
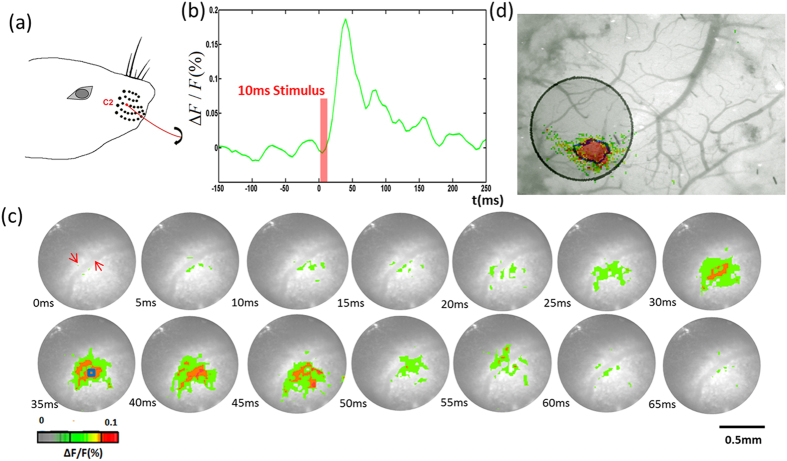
(**a**) C2 whisker stimulation. (**b**) Change in fluorescence (ΔF/F(%), ordinate) in response to stimulation. Fluorescence signal was calculated from the ROI (small blue square: 5 by 5 pixels) shown in Fig. 1(c) at 35 ms post-stimulation. (**c**) Voltage-sensitive dye optical images showing single-whisker (C2) stimulation fluorescence changes in the cortex. The stimulus onset was 0 ms. Time period after stimulation is indicated at the bottom left corner of each image. (**d**) The same area with the GRIN lens superimposed with image taken by conventional system. The black circle is the field view of GRIN lens and the black contour is the boundary of the activated area imaged by GRIN lens.

First we carried out an experiment to record the cortical signal corresponding to single whisker stimulation using our GRIN-rod-lens VSDi system. The purpose of this experiment was to confirm the performance of the needle-based imaging system. For imaging, the distal end of the GRIN rod lens was positioned above the recording area and directed such that its optical axis was perpendicular to the cranial window. The focusing plane was set to 300 μm below the dural surface. As illustrated in Fig. 1(a), C2 whisker was stimulated for a period of 10 ms. The signal appeared 20 ms after the stimulus onset, and reached its peak at about 40 ms after the stimulus onset. After reaching the maximum value, the activated signals decreased until returning to baseline [Fig. 1(b)]. The changes in activation area followed a pattern corresponding to the stimulation [Fig. 1(c)]. Blood vessels were visualized due to different light absorption of the hemoglobin and the cortical tissue (labeled in red arrow in Fig. 1(c)). The results of cortical signal recording agreed well with the conventional VSDi system without GRIN rod lens, indicating that the GRIN-rod-lens imaging system is suitable for VSDi signal recording^24^. In addition, we imaged the cortex first with the conventional system and then the same area with the GRIN-rod-lens for comparison. As illustrated in Fig. 1(d), there was no notable difference in the boundary of the activity patterns.

### Signal recording in the thalamus

#### Mapping VPM responses to stimulation of single whisker.

**Figure 2:**
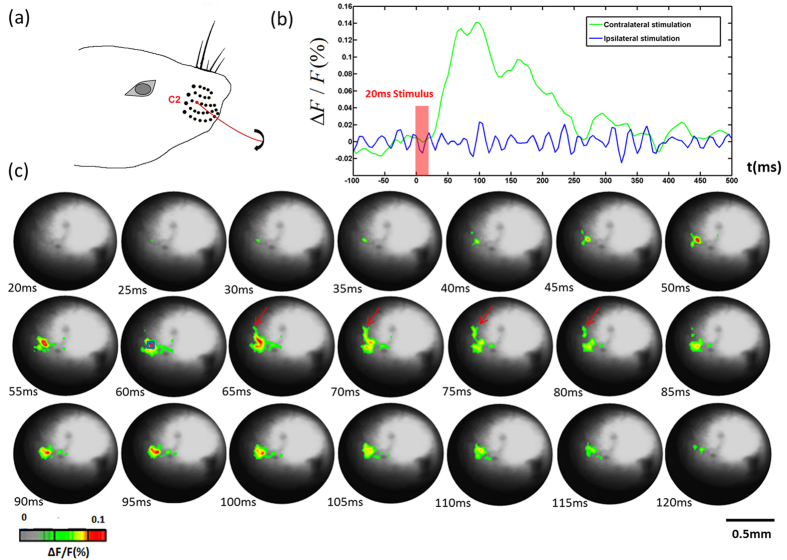
(**a**) C2 whisker for stimulation. (**b**) Changes in fluorescence (ΔF/F(%), ordinate) in response to C2 whisker stimulation. Fluorescence signal was recorded from the small blue square (5 by 5 pixels) marked on the image shown in Fig. 2(c) at 60 ms. (**c**) Voltage-sensitive dye optical images showing single-whisker (C2) stimulation fluorescence changes in the thalamus. Time period after stimulation is indicated at the bottom left corner of each image.

Fig. 2(a) depictss C2 whisker stimulation. Fig. 2(b) shows changes in fluorescence (ΔF/F) in response to C2 whisker stimulation. Ipsilateral stimulation data was acquired as control. Fig. 2(c) shows the GRIN rod lens images of changes in fluorescence signals in response to contralateral C2 whisker stimulation. The signal appeared about 20 ms after the stimulus onset, and reached its peak at 55–60 ms after the stimulus onset. An interesting observation is that after reaching its maximum value, the activated areas started to spread at 65 ms after the stimulus onset (indicated by the red arrows in Fig. 2(c)). Another peak appeared at 95 ms, which may be due to the whisker swinging back after the 10-ms air puff stimulation. After 110 ms, the activated signals disappeared gradually.

#### Mapping responses to stimulation of multiple whiskers

**Figure 3:**
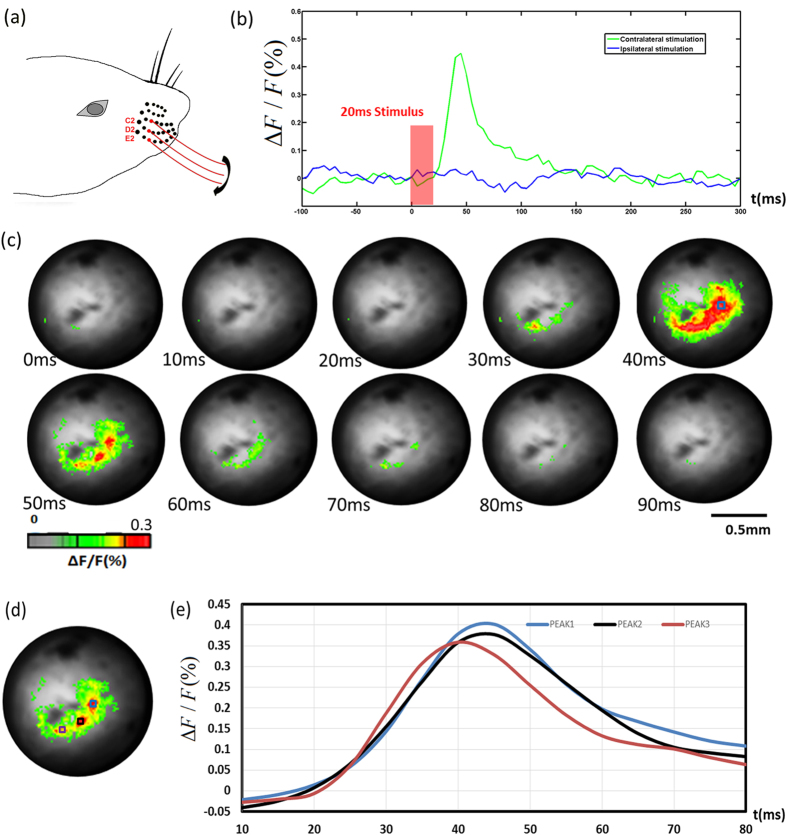
(**a**) C2, D2 and E2 whiskers for stimulation; (**b**) Changes in fluorescence (ΔF/F(%), ordinate) in response to whiskers stimulation. Fluorescence signal was recorded in the small blue square (5 by 5 pixels) marked on the image shown in Fig. 3(c) at 40 ms. (**c**) Voltage-sensitive dye optical images showing three whiskers stimulation fluorescence changes in thalamus. Time period after stimulation is indicated at the bottom left corner of each image. (**d**) Three areas chosen to plot the time course. (**e**) Changes in fluorescence (ΔF/F(%), ordinate) in response to whiskers stimulation. Fluorescence signal was recorded in the small squares marked on Fig. 3(d).

**Figure 4:**
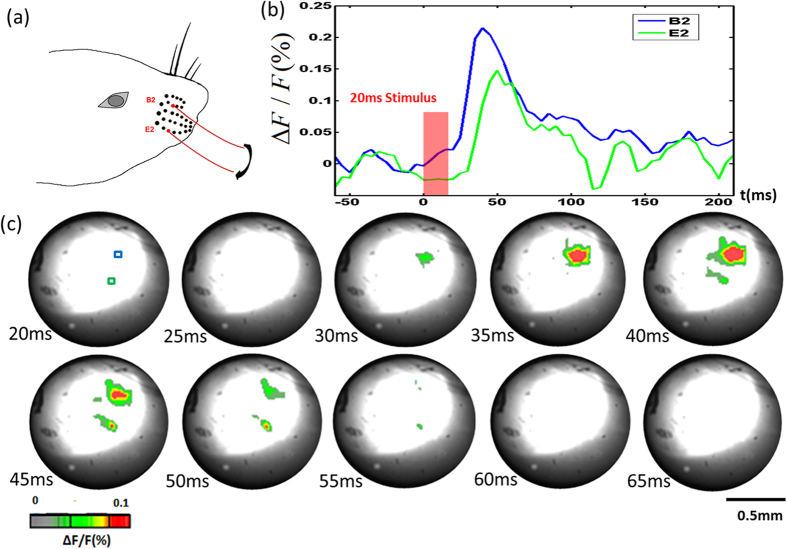
(**a**) B2 and E2 whisker stimulation. (**b**) Changes in fluorescence (ΔF/F(%), ordinate) in response to whiskers stimulation. Fluorescence signal was recorded in the small blue and green squares (5 by 5 pixels) marked on the image shown in Fig. 4(c) at 20 ms. (**c**) Voltage-sensitive dye optical images showing two whisker stimulation fluorescence changes in the thalamus. Time period after stimulation is indicated at the bottom left corner of each image.

To further investigate whether the needle-imaging system could differentiate multiple whisker-evoked responses in the VPM, we performed a three-whisker stimulation experiment. C2, D2 and E2 whiskers were stimulated at the same time [Fig. 3(a)]. Ipsilateral stimulation data was acquired as control [Fig. 3(b)]. Fig. 3(c) shows the signal appearance and diminishing in both activation area size and signal magnitude. At 50 ms, we can clearly see the three areas, which correspond to three different whiskers. In order to see the temporal response more closely, signals from the three “hot spots” areas^28^,^29^,^30^ were plotted in Fig. 3(d). There was a clear 5 ms peak delay between one area and the other two that may be due to the different whiskers lengths. Fig. 4 shows the result of such an experiment in which whiskers B2 and E2 were stimulated at nearly the same time. B2 and E2 whiskers have a larger spatial separation on the snout. In Fig. 4(c), we can clearly see two separate responses. There was also a clear peak delay of 10 ms between these two responses.

#### Mapping responses to stimulation of multiple whiskers at different times.

To further investigate whether the imaging system could differentiate the responses in the VPM when whiskers are stimulated at different times, we performed a time-difference stimulation experiment.

**Figure 5:**
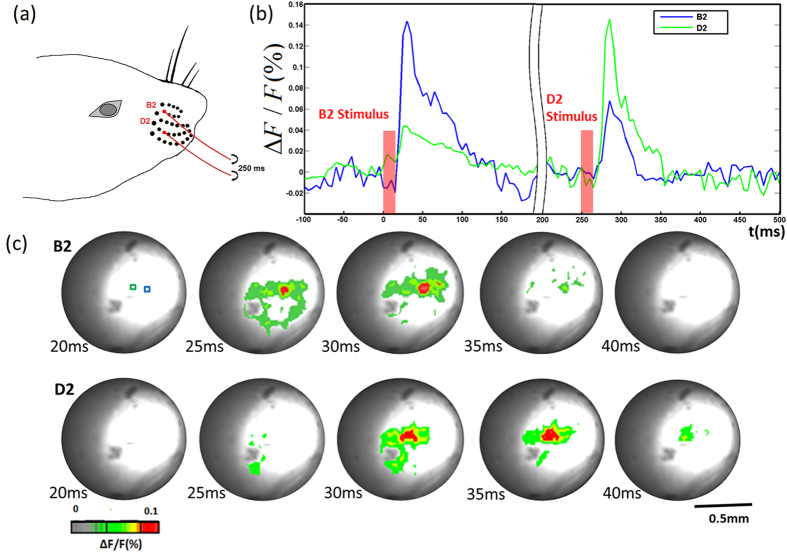
(**a**) B2 and D2 whisker stimulation; (**b**) Change in fluorescence (ΔF/F(%), ordinate) in response to whiskers stimulation. Fluorescence signal was recorded in the small blue and green squares (5 by 5 pixels) marked in Fig. 5(c), B2, 20 ms; (**c**) Voltage-sensitive dye optical images showing whisker (B2 and D2) stimulation fluorescence changes in the thalamus. Time period after stimulation is indicated at the bottom left corner of each image. The first row showing fluorescence response to B2 stimulation, the second row showing fluorescence response to D2 stimulation which was set 250 ms after B2 stimulation.

B2 and D2 whiskers were used for stimulation [Fig. 5(a)]. Stimulation for B2 was set at 70^th^ frame (350 ms) and stimulation for D2 was set at 120^th^ frame (600 ms). The first response to B2 reached its maximum at 30 ms after B2 whisker stimulation, and the second response to D2 whisker reached its maximum at 35 ms after D2 whisker stimulation as shown in Fig. 5(b). The centers of the two response areas are separated by approximately 150 μm [Fig. 5(c)], which agreed well with the anatomy^31^.

#### Exploring the effect of corticothalamic inputs to activities in thalamic barreloids

**Figure 6:**
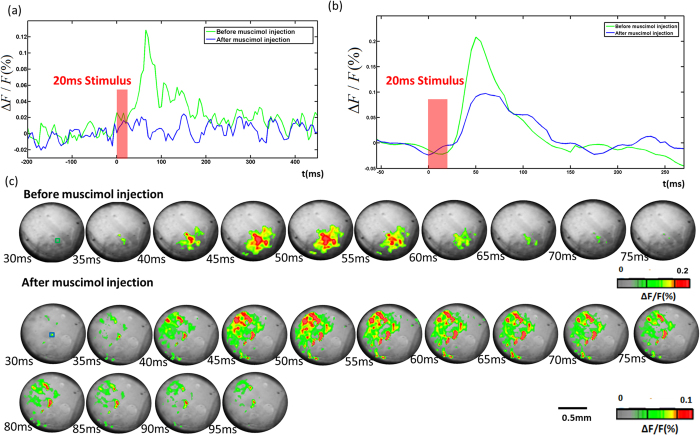
(**a**) Changes in fluorescence (ΔF/F(%), ordinate) in response to C2 whisker stimulation of the same area (5 by 5 pixels) in cortex before and after muscimol injection. (**b**) Changes in fluorescence (ΔF/F(%), ordinate) in response to C2 whisker stimulation of the same area (5 by 5 pixels) in VPM before and after muscimol injection. Fluorescence signal was recorded from the small blue square marked on the image shown in Fig. 6(c) at 30 ms. (**c**) Voltage-sensitive dye optical images showing single-whisker (C2) stimulation fluorescence changes in thalamus before and after muscimol injection. Time period after stimulation is indicated at the bottom left corner of each image. Different color maps were shown at the bottom of each images sequences.

**Figure 7:**
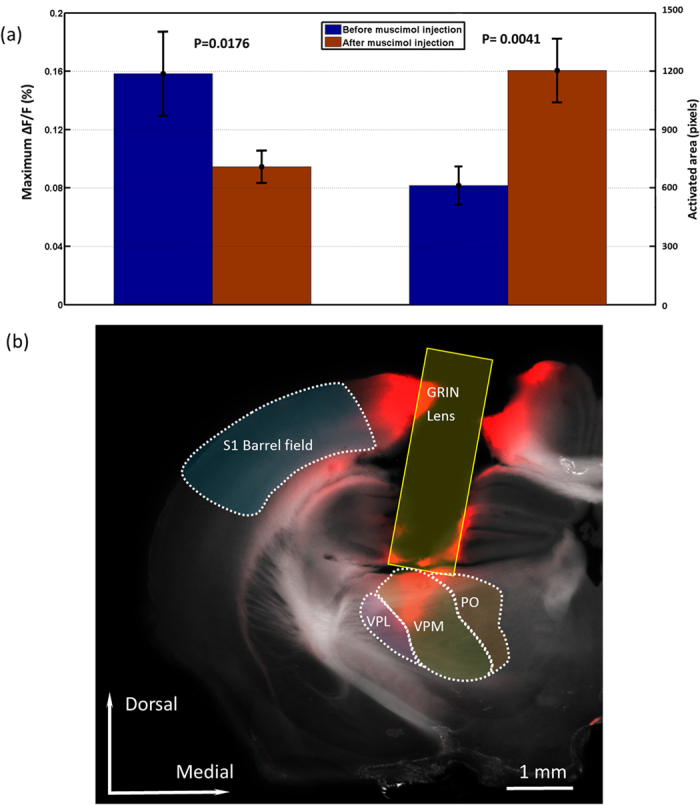
(**a**)Statistics of signal amplitudes and activated areas in VPM before and after muscimol injection. The signal amplitude of the group with muscimol injection (n = 3, from 3 different animals) was statistically (P < 0.05) smaller compared to control group (without muscimol injection: n = 4, from 4 different animals). The activated area in the group with muscimol injection was statistically (P < 0.05) larger than control group. (**b**) Brain slice taken 2.0 mm posterior from bregma and superimposed with the brain atlas^50^ (outlined and labeled areas). VPM—ventral posterior medial nucleus, PO—posteromedial thalamic nucleus, VPl—ventral posterolateral thalamic nucleus, S1- primary somatosensory cortex. Red color indicates RH-1691 dye fluorescence spread from the track of the GRIN probe and RH-1691 injection.

We performed imaging experiments in the thalamus of 4 mice following muscimol injection into the barrel cortex. Blocking cortical activity abolishes VSD signal in the cortex but not in the VPM. This is exciting new results which allows one to compare thalamic activation in the absence of corticothalamic inputs or when the cortex is silenced. Fig. 6(a) shows wave plot of the changes in fluorescence (ΔF/F(%), ordinate) in response to C2 whisker stimulation of the same area (5 by 5 pixels) in cortex before and after muscimol injection. We can clearly see that the responses in the cortex were inhibited by muscimol. In the following experiment, we first inserted the GRIN rod lens to the VPM based on the same procedure described above. Then the fluorescence signals in VPM before and after muscimol injection in response to contralateral C2 whisker stimulation were acquired as shown in Fig. 6(c). Compared to the signal without silencing the cortex, we observed that the activation area is much wider and scattered. Fig. 6(b) shows changes in fluorescence (ΔF/F(%), ordinate) in response to C2 whisker stimulation before and after muscimol injection. Fluorescence signal was recorded from the small blue and green squares marked on the images shown in Fig. 6(c) at 30 ms. We can see the signal magnitude is relatively low compared to the signal magnitude before muscimol injection. To quantify the effect of corticothalamic inputs to activities in the thalamic barreloids, the signal amplitude and activated area during stimulation were determined as shown in Fig. 7(a). The group with muscimol injection consisted of 3 data sets from 3 different animals. Control experiments (without muscimol injection) were performed on 4 data sets from 4 different animals. The signal amplitude in the group with muscimol injection was statistically smaller compared with the control group (P = 0.0176). And activated area in the group with muscimol injection was nearly twice wide as the control group (P = 0.0041). We conclude that our data may provide evidence indicating that feedback from the cortex plays a crucial role in shaping thalamic responses^32^.

Fig. 7(b) shows the GRIN rod lens track to the VPM in brain slice. Red color indicates RH-1691 dye fluorescence spread from the track of the GRIN probe. The positions of VPL,VPM and PO are labeled. While the GRIN rod lens causes a large area of damage to the brain, the barrel field of the somatosensory cortex and thalamocortical pathway are spared. This is mainly due to an angled approach to the VPM medially from the cortex and hippocampus. We can see the dye spread to VPM clearly and the GRIN rod lens is right above the VPM, which indicates that the stereotaxic coordinates we used allowed for precise location of the VPM.

## Discussion

Brain optical imaging has developed considerably within the last few decades^2^. Optical methods can offer both high spatial and temporal resolutions and are therefore particularly promising for measuring the hemodynamic, metabolic, and neuronal activity *in vivo*^7^,^8^,^13^,^33^,^34^. VSDi, which utilizes a high-speed CCD camera, offers an opportunity to study the activity of neuronal ensembles *in vivo* with relatively high spatial (up to 20 μm) and temporal resolution (up to few milliseconds, which is comparable to electrophysiology)^35^,^36^. However, since CCD cameras integrate the back-scattered light from various depths, VSDi cannot detect depth-resolved functional activation^37^. In contrast with the single photon imaging, multi-photon microscopy has been used for functional neuronal imaging, and recent developments extend the penetration depths up to 1.6 mm in a mouse^33^. On the other hand, light penetration is fundamentally limited by scattering in the tissue. To overcome this limitation, several groups explored optical endoscopes. Miniature optical endoscopes are typically based on commercially available gradient-index (GRIN) rod lenses or imaging fiber bundles. GRIN rod lenses, which are typically 350–2,000 μm in diameter, can provide relatively high resolution, and have been used in deep brain imaging with relatively little injury^27^,^38^,^39^,^40^,^41^,^42^,^43^.

Many studies investigated information processing along the whisker-barrel system, using electrophysiological and morphological techniques. However, imaging studies in normal laboratory rodents and transgenic mice have been limited to the barrel cortex due to accessibility issues^24^,^35^,^44^,^45^,^46^,^47^. Light penetration and scattering limits the usage of the optical imaging methods in subcortical structures. Our present *in vivo* results show that the combination of VSDi and GRIN optical probe can be utilized in imaging deep brain structures and can be adopted for use in freely moving animals through flexible imaging fiber bundle^48^.

It is important to note the limitations of VSDi imaging at multiple levels of a sensory system. A notable one is the temporal resolution. The temporal resolution of the system we used is 5 ms, which is much longer than synaptic relays from the brainstem to cortex. Thus, we were not surprised that the latency of the VSDi signal, obtained from the VPM, and the time courses were similar for the optic signals recorded from the barrel cortex. In this regard, VSDi approach is not as sensitive as standard electrophysiological recording techniques because of the limited temporal resolution. The time differences between neural responses in the VPM and the barrel field can be just few milliseconds, which is shorter than our temporal resolution.

The VSDI signal and the physical relationship to barreloids in each experiment is not feasible at the moment. However if we express GFP or other fluorescent tags in barreloid neurons and activate them we might be able to pick up individual barreloids and specific activity patterns in relation to them. At the present time we can only compare the size of the activated areas in the VPM with respect to stained brain sections after imaging. Another limitation in imaging from deep brain structures is that the barreloids in the VPM are not all in the same plane from the tip of the GRIN-lens. The axes of the barreloids rows are not orthogonally located to the focal plane, and many of them are located slightly above or below the focus. Thus, the system we used does not have depth resolution for three-dimensional (3-D) neuronal maps. Lastly, the source of the fluorescence also has its own 3D structure located at an undetermined distance from the focal plane is projected as 2D optical patterns. 3D imaging through GRIN-rod-lens needle microscope is also feasible using advanced image reconstruction algorithms^49^. Nonetheless, the compact size and functionality of GRIN optical devices in combination with VSDi are enabling imaging deep brain structures and functions in the mammalian brain *in vivo*.

## Methods

The experimental procedures were all in accordance with the National Institute of Health guidelines for the care of experimental animals (National Institute of Health, Committee on Care and Use of Laboratory Animals, 1996) and the Animal Use and Care Committee of the University of Maryland approved all experimental protocols.

### Animal preparation

We imaged voltage-sensitive dye optical signals in eight adult mice (B6 male and female, 20–30 g body weight, age 5–10 weeks). All animals were anesthetized with urethane (1.15 g/kg) with body temperature maintained at 37 °C with a heating blanket. The animal’s head was placed in a stereotaxic frame. For surgical preparation, the head was shaved, and a midline cutaneous incision was made. The skin over the skull was retracted, and a cranial window, 1.5 mm in diameter, was made using a dental drill^1^. A 10 μL Hamilton syringe, outer needle diameter 0.3 mm, was used to inject 0.3–0.5 μL voltage-sensitive dye RH-1691 (Optical Imaging Ltd, 1.0 mg/mL in the artificial cerebrospinal fluid (ACSF)) into the VPM using stereotaxic coordinates (−1.7 posterior, 1.6 lateral and 3.1 mm below dura mater )^50^ over 5 minutes. The cranial opening was covered by a drop of high viscosity silicone oil to prevent drying and decrease brain pulsation^35^. Imaging was started 15–20 minutes after dye loading.

### Experimental setup

**Figure 8:**
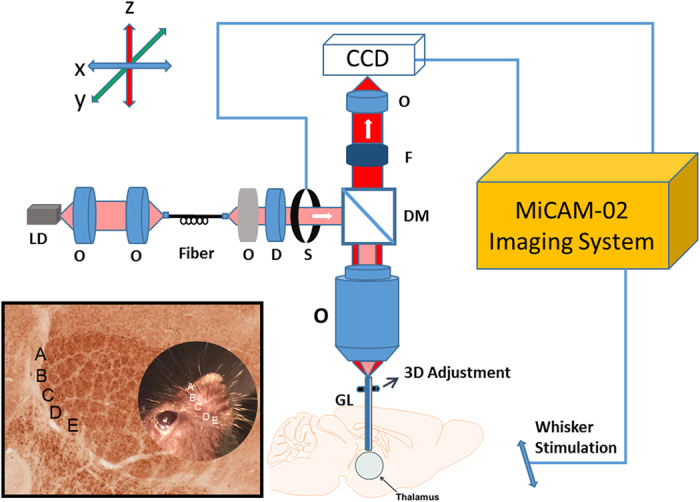
Schematic of the VSDi needle system. O: objective lens; S: shutter; D: diffuser; F: filter; DM: dichroic mirror; GL: GRIN rod lens. Bottom left inset shows barreloid organization with respect to the whisker pad (circled photo). Cytochrome oxidase stained section, dorsal is up, lateral is to the left. Whisker rows A–E are indicated.

Figure 8 illustrates the schematic of the needle-based VSDi imaging system. The system is equipped with a 1-mm diameter GRIN rod lens (NA 0.113; Go Foton Corporation), which provides an imaging field-of-view (FOV) of ~0.9 mm and relays the image from the mouse brain at the distal end of the needle probe back to the focal plane of the objective (Leica Objective Planapo 2.0 ×, M-series). The system utilizes a 637 nm laser diode as its light source, which is coupled with a single-mode fiber to shape its light beam. A diffuser is used to make the light more uniform. The light is collimated by an objective and goes through a dichroic mirror (650 nm, single edge dichroic beam splitter; FF650-DiO1-50 × 70 mm; Andover Corporation). A shutter is applied to control the excitation light state and avoid dye photobleaching. The light is then coupled to the GRIN rod lens by the microscope objective. A custom-built motorized 3D micro-stage facilitates accurate light coupling between the objective and GRIN rod lens. The emitted fluorescent light is collected back through the GRIN rod lens, objective, dichroic mirror, an emission filter (695 nm, 695FG07-50, Andover Corporation), and finally imaged on a high-speed CCD camera (MiCAM02-HR, SciMedia, Ltd). For deep tissue imaging, after aligning the GRIN rod lens to the objective, the GRIN-rod-lens probe was gently inserted into the anesthetized mouse thalamus by moving up the animal stage slowly and using stereotaxic coordinates^50^.

### Stimuli and data acquisition

Each experimental session consisted of 10–30 trials, with 200 frames per trial. Data acquisition rate was 5 ms/frame (200 Hz). For single stimulus experiments, the stimulus (whisker deflection) was presented at the 100^th^ frame (one stimulus per trial). For dual stimulus experiment, the two stimuli were set at the 70^th^ frame and the 120^th^ frame, respectively. The pause between trials was 10 seconds. Fluorescence changes were calculated as ΔF/F (%) in the recording area using Brain Vision Analyzer (Brain Vision Inc., Tokyo, Japan). Before stimulation, all whiskers, with the exception of the designated whiskers used for experiment, were clipped close to the skin. To perform stimulation, a glass pipette (1.0 mm in diameter) fitted on an XYZ manipulator was aimed at the designated whisker. Air-puff stimulus (duration 10–20 ms) was applied through a Picospritzer pressure valve connected to the glass pipette^35^. The Picospritzer was coupled to the imaging system through the MiCAM-02 controller, so the air could be puffed onto the whiskers at precisely controlled time points. Trains of whisker stimuli were delivered and the associated changes in fluorescence signals were recorded in the contralateral thalamus. In addition, we also performed ipsilateral stimulation as control^21^. In some experiments muscimol (0.1 μl, 10 mM in ACSF), a selective GABA_A_ receptor agonist, was injected at a depth of 300 μm below the cortical surface^51^. The injection was performed with a glass pipette (20 μm tip diameter) attached to a Nanojet II injector (Drummond Scientific, USA)^52^

### Data analysis

For single stimulation experiments, the final ten pre-stimulus frames (i.e., 90–99^th^ frame) were averaged as the baseline image. The baseline image was then subtracted from each subsequent frame to obtain changes in fluorescence signals. For dual stimuli experiments, the final ten pre-stimulus frames, i.e., 60–69^th^ frame and 110–119^th^ frame were averaged as the baseline for the 1^st^ and 2^nd^ stimulus, respectively. Pixels which exhibited a change in fluorescence (ΔF/F) greater than 50% of the maximum change were identified as activated regions^24^. Subsequently, we obtained pseudocolor maps of the areas activated by whisker stimulation. In the experiment exploring the effect of corticothalamic inputs, obtained data were expressed as mean ± standard deviation. Statistical analyses were then carried out using MATLAB. Student t-test was used to compare the signal amplitude and activated area (pixel number) in VPM before and after muscimol injection. P < 0.05 was considered to indicate a statistically significant difference.

### Histology

After the experiment, the animal was euthanized by barbiturate overdose and perfused by 4% paraformaldehyde and decapitated. The brain was extracted and sliced coronally (0.3 mm thickness) using a vibratome. The slice was photographed using fluorescence microscope. The probe track was superimposed^50^.

## Additional Information

**How to cite this article**: Tang, Q. *et al. In Vivo* Voltage-Sensitive Dye Imaging of Subcortical Brain Function. *Sci. Rep.* **5**, 17325; doi: 10.1038/srep17325 (2015).
